# FLAVIdB: A data mining system for knowledge discovery in flaviviruses with direct applications in immunology and vaccinology

**Published:** 2011

**Authors:** Lars Rønn Olsen, Guang Lan Zhang, Ellis L. Reinherz, Vladimir Brusic

**Affiliations:** 1 Cancer Vaccine Center, Dana-Farber Cancer Institute, Harvard Medical School, Boston, MA 02115, USA; 2 Laboratory of Immunobiology, Dana-Farber Cancer Institute, Boston, MA 02115, USA; 3 Department of Medicine, Harvard Medical School, Boston, MA 02115, USA; 4 Center for Biological Sequence Analysis, Department of Systems Biology, Technical University of Denmark, Lyngby, Denmark

## Abstract

**Background:**

The *flavivirus* genus is unusually large, comprising more than 70 species, of which more than half are known human pathogens. It includes a set of clinically relevant infectious agents such as dengue, West Nile, yellow fever, and Japanese encephalitis viruses. Although these pathogens have been studied extensively, safe and efficient vaccines lack for the majority of the flaviviruses.

**Results:**

We have assembled a database that combines antigenic data of flaviviruses, specialized analysis tools, and workflows for automated complex analyses focusing on applications in immunology and vaccinology. FLAVIdB contains 12,858 entries of flavivirus antigen sequences, 184 verified T-cell epitopes, 201 verified B-cell epitopes, and 4 representative molecular structures of the dengue virus envelope protein. FLAVIdB was assembled by collection, annotation, and integration of data from GenBank, GenPept, UniProt, IEDB, and PDB. The data were subject to extensive quality control (redundancy elimination, error detection, and vocabulary consolidation). Further annotation of selected functionally relevant features was performed by organizing information extracted from the literature. The database was incorporated into a web-accessible data mining system, combining specialized data analysis tools for integrated analysis of relevant data categories (protein sequences, macromolecular structures, and immune epitopes). The data mining system includes tools for variability and conservation analysis, T-cell epitope prediction, and characterization of neutralizing components of B-cell epitopes. FLAVIdB is accessible at cvc.dfci.harvard.edu/flavi/

**Conclusion:**

FLAVIdB represents a new generation of databases in which data and tools are integrated into a data mining infrastructures specifically designed to aid rational vaccine design by discovery of vaccine targets.

## Background

More than 70 known viral species belong to the flavivirus genus. The flavivirus genus can be divided into three clusters, fourteen clades, and 70 species [1]. The clusters are based on host-vector association: mosquito-borne, tick-borne, and no-vector viruses. The members of flavivirus clades share >69% pairwise nucleotide sequence identity, while members of individual species share >84% identity [1]. More than half of these single-stranded RNA viruses are known human pathogens [2]. The most important human pathogens among flaviviruses are West Nile virus (WNV), dengue virus (DENV), Tick-borne encephalitis virus (TBEV), Japanese encephalitis virus encephalitis virus (JEV), and yellow fever virus (YFV). Flaviviruses pose a significant global public health threat since they are responsible for hundreds of millions of human infections each year. Hailed as one of the most successful vaccines ever developed, the live attenuated YFV vaccine [3–4] however often induces severe adverse effects [5], thus leaving room for significant improvements. Safe and efficient JEV and TBEV vaccines have also been developed, although these have limited global application due to high production prices [6]. To date, successful vaccines against DENV, WNV, and a range of other emerging flavivirus pathogens have proven elusive.

Because of the high sequence similarity between the flavivirus species, the analysis of antigenic diversity, both intra- and inter-species, offers important insights in potential cross-reactivity, cross-protection following infection or immunization, and clues for understanding the factors that determine disease severity. Cross-reactivity and cross-protection is particularly relevant in the case of DENV, due to issues relating to severe consequences of secondary infection. While the factors that lead to severe dengue disease are unclear, it was proposed that it is related to misdirected immune responses including antibody dependent enhancement (ADE) [7] as well as exaggerated and partially misdirected T-cell responses [8].

Sequencing efforts in recent years have produced a large body of flavivirus molecular data enabling advanced data analyses for rational vaccine design. Primary databases such as GenBank [9] contain comprehensive collections of nucleotide sequence data. Protein sequence data are available from UniProt knowledgebase [10] which offers curated, high quality protein data. A number of dedicated flavivirus databases are available such as Flavitrack [11] and the NIAID Virus Pathogen Database and Analysis Resource (ViPR) (www.viprbrc.org). Both Flavitrack and ViPR provide access to curated sequence data and they include a selection of sequence analysis tools. Particularly ViPR offers an abundance of useful analysis tools such as sequence similarity search, multiple sequence alignment (MSA), single nucleotide polymorphism (SNP) analysis, and construction of phylogenetic trees, neatly organized in their workbench analysis environment. However, data mining for vaccine target discovery requires complex database search requests and often a combination of several different tools (for example, prediction of epitopes is often preceded by extensive conservation and variability analysis) integrated in data mining systems for automated knowledge extraction and knowledge discovery.

To aid the analysis of immunological properties and discovery of vaccine targets in the flaviviruses, we constructed the FLAVIdB – a database of *Flavivirus* spp. that contains information on protein sequences, immunological data, and structural data. These data are integrated into a modular extensible infrastructure that enables detailed analysis of sequences and their antigenic properties through application of data mining techniques [12]. The tools can be applied individually or by using predefined workflows designed for discovery of vaccine targets.

## Methods

### Data collection

**Figure 1:**
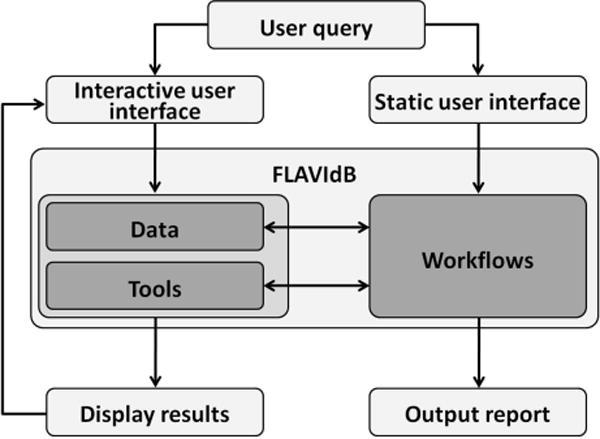
Summary of the structure of FLAVIdB. Users can access FLAVIdB through the interactive interface for direct access to data or tools, or through static interface and predefined workflows. Workflows use a predefined process to access data and tools and to produce a report.

The sequence data for FLAVIdB were extracted from primary sources GenBank, GenPept [9] and UniProt [14]. The raw data were downloaded for species in the *Flavivirus* genus (NCBI taxonomy ID: 11051), and subsequently transformed into an XML format. Data module of experimentally determined B- and HLA class I and II T-cell epitopes was populated with data extracted from IEDB [15] as well as additional epitope data retrieved from the literature. The epitope data were enriched with data from binding assays, neutralization assays, and cross -protective properties. Macromolecular structure data from protein data bank (PDB) [16] was also extracted for the envelope proteins of DENV. The content of FLAVIdB is searchable using keyword search and is available for download by users. The main purposes of FLAVIdB are data integration, data mining, and knowledge extraction for applications in immunology and vaccinology. The overall framework of the system is shown in Figure 1.

### Data cleaning

Sequences that had duplicate entries were merged into single entries to minimize data redundancy. In the primary sources some of the available sequences were well-annotated, some had incomplete annotations, whereas others lacked annotation altogether. The NCBI GenPept protein reference sequences served as templates for annotation of viral protein sequences. For quality control, of existing protein annotations and the addition of missing annotations, query sequences were aligned to the appropriate reference sequence using MAFFT MSA tool [17]. Sequence fragments shorter than 23 amino acids were not included in the database. The threshold 23 was chosen because it is the length of the shortest protein naturally occurring in the *Flavivirus* proteome (the 2K peptide). Existing positional annotations were compared to the MSA and corrected when needed. The missing positional annotations were generated from MSA positions and included in FLAVIdB records.

### Data enrichment

Journal publications corresponding to the strain entries were extracted from PubMed (www.ncbi.nlm.nih.gov/pubmed) when available. Semi-automated extraction of articles was performed to retrieve information missing in the GenBank entries, but otherwise available. Manual checking was necessary because of limited extent of standardized fields, nomenclature, and terms in primary sources.

### Basic search tools

#### Keyword search

Beyond the basic utility of keyword search, FLAVIdB also offers options for filtering data based on species, pathology (disease outcome or fatal/non-fatal), proteins, strain type (wild type, laboratory strain, or vaccine strain), entry type (complete proteomes or partial proteomes), and host. The data retrieval function also serves as a tool for selection of subsets of sequences for comparative analyses.

#### Sequence similarity search

Sequence similarity search of the FLAVIdB can be performed using the basic local alignment search tool (BLAST) algorithm [18] through an integrated BLAST module within the FLAVIdB. We recommend that integrated BLAST tool is used with the default parameters, while for advanced users there is an option to set different values for parameters such as E value, word size, substitution matrix, and gap cost.

### Basic analysis tools

#### Multiple sequence alignment

The MSA can be performed for three or more sequences in FLAVIdB using the MAFFT tool [17]. The output is color coded for easy visualization of variations, matches, and gap insertions in the alignment. The search interface enales a selection of pre-defined subsets by virus type or subtype, pathology, individual protein, strain type (wild type, laboratory, or vaccine), size (complete or partial), or host of isolation. Furthermore, MSA can be performed on selected results from BLAST search.

#### Sequence conservation and variability metrics

FLAVIdB is equipped with the tools for sequence conservation and variability analysis. Variability analysis can be performed on entries grouped by protein and further narrowed down by virus type or subtype, and by host of isolation. The variability analysis at amino acid level is based on calculation of Shannon entropy [19] at each position in a MSA. The entropy is calculated using the formula:

(1) $$H(x)=-\sum_{i=1}^{I}P_{i}(x)\log_{2}(P_{i}(x))$$

where *H* is the entropy, *x* is the position in the MSA, *i* represent individual amino acids at position *x*, *I* is the number of different amino acids on position *x*, and *Pi* is the frequency of the given amino acid. Conservation of a position, *x*, is defined by the frequency of the consensus amino acid.

#### Block entropy analysis

To accommodate conservation and variability analysis of T-cell epitopes, we developed a new entropy measurement method, specifically designed for the analysis of short peptides, 8–11 amino acids in length. This approach is based on calculation of entropy for each overlapping window (block) of 8–11 amino acids in length in a given MSA of homologous proteins. Then, entropy and conservation is calculated for the peptides, rather than individual residues. Since T-cell epitopes are recognized as peptides and not as individual residues, this approach provides a more representative image of the conservation of linear epitopes. For class I T-cell epitopes, the size of the window ranges between 8 and 11 amino acids [20] while class II epitopes are typically 13–20 amino acids long with a binding core of minimum 9 amino acids long [21–22]. The results of block entropy analysis are displayed with traditional entropy analysis to further clarify the peptide diversity and its relationship to individual amino acid diversity.

#### Species classification

FLAVIdB enables classification of newly acquired sequences that belong to species of flaviviruses. Classification of species to unassigned strains is performed using the BLAST algorithm [18] in combination with the knowledge of phylogenetic traits of the genus *flavivirus* reported in [1]. Because its main purpose is the analysis of antigenic properties of viruses, FLAVIdB has nucleotide sequence data for each of the protein entries in the background and not searchable by keywords or by browsing. For species classification, we use the similarity rule defined in [1]. The nucleotide sequence similarity search is performed in FLAVIdB using BLAST algorithm, after which the query is compared to the highest scoring match. If the pairwise identity of query and match is 84% or greater, the query is considered of the same species as its match. Species classification can only be performed on full (or nearly full) viral genome sequences. Since some proteins are far less variable than others, submitting a single gene sequences could give ambiguous or incorrect results.

### Advanced analysis tools

#### Prediction of MHC class I binders

Prediction of peptide binding affinity to MHC class I is performed using the neural network and weight matrix based prediction algorithm; NetMHC 3.2 [23]. Epitope prediction in FLAVIdB is available for the following HLA alleles: HLA-A*0201, HLA-A*0301, HLA-A*1101, HLA-A*2402, HLA-B*0702, HLA-B*0801, and HLA-B*1501, since NetMHC 3.2 predictions for these alleles were independently validated and assessed as highly accurate [24].

#### Prediction of MHC class II binders

Prediction of peptide binding affinity to MHC class II is performed using NetMHCIIpan 2.1 [25] for the alleles HLA-DRB1*0101, HLA-DRB1*1101, HLA-DRB1*0401, and HLA-DRB1*0701 for which prediction of binding affinities were independently validated and assessed as highly accurate [26].

#### Characterization of shared neutralizing components of B-cell epitopes (BBscore)

In DENV, it is essential that antibody based vaccines afford broad neutralization across all four serotypes. The tool for B-cell epitope analysis in FLAVIdB is based on comparative analysis of known B-cell epitopes together with comparison of corresponding binding and neutralization assay data. Features shared by neutralizing epitopes against all four serotypes are extracted and presented on 3D models of the envelope protein. Furthermore, users can map these shared features onto any envelope protein sequence in FLAVIdB. At present, structural data for the envelope protein is only publically available (in the pdb database) for dengue, West Nile, Japanese encephalitis, Langat, Omsk hemhorraghic fever, and yellow fever viruses. Useful amounts of experimentally validated epitopes and corresponding biochemical/functional assay data are only available for DENV and WNV. Thus, the BBscore tool is currently limited to the analysis of DENV and WNV. The output of this analysis can automatically improve both the breadth and depth of characterization of neutralizing properties of antigenic sites on surface proteins as more epitopes and assay data becomes available in primary sources such as the IEDB [15].

## Results and Discussion

### Database content

As of June 2011, FLAVIdB contains 12,858 entries, with sequences from 87 flavivirus species consisting of 65 classified species and 24 provisional or unclassified species (see Table 1)

The first release of the database (June, 2011) has 3,120 complete proteome sequences and 9,738 partial sequences. Each proteome entry was annotated as its individual protein (or in some cases, protein fragment) constituents. Each entry contains protein sequences along with additional annotations describing various strain information (see Table 2).

Each entry was given a shorthand nomenclature, sequence name and source strain identifier. The nomenclature contains information about species, host, country (ISO code), strain name, isolate name, clone name, and year of collection. An example of a FLAVIdB nomenclature is:

DENV1|Human|TH|NIID2|133|02−20|2002

An entry with this nomenclature represents a DENV type 1 isolated from a human host, at geographic location Thailand, in year 2002, with the specific strain NIID2, isolate 133, clone 02–20.

For the repository of experimentally determined B-cell epitopes, each entry (only applicable to DENV in the current release) describes positions in protein sequence, species, serotype, publication reference, and data from binding assays and neutralization assays. For the repository of experimentally determined T-cell epitopes, each entry is described by location in protein, epitope sequence, HLA restriction, and publication references.

### Data quality

The population of FLAVIdB was subject to a rigorous quality control. Approximately 500 sequence errors and artifacts (nonsensical characters, frameshift mutations rendering the protein sequences in question of no use to conservation and variability analysis) were detected and corrected or removed. More than 1,000 metadata terms used in primary sources were consolidated into approximately 200. To support data term consolidation, a library of all fields from all entries was created. The library was used for semi-automated consolidating the entry vocabulary by merging redundancies such as “US”, “U.S.”, “United States of America”, “America”, etc., into the FLAVIdB convention: “USA” and the corresponding ISO code used in FLAVIdB nomenclature, “US”. The species classification error analysis led to the identification and correction of 17 strains, and the classification of seven previously unclassified serotypes of DENV. Furthermore, the entries were enriched by definition of additional metadata. The specific information includes: location of collection, host of collection, time of collection, strain type (whether entry genome was derived from wild type, laboratory strain, or vaccine strain), pathology (whether infection led to disease and/or fatality) and comments on protein function (some mutant strains encode nonfunctional protein products). The differences between the available annotations in the primary data from external sources and the enriched data in FLAVIdB are shown in Table 3.

### Analysis tools

FLAVIdB is equipped with several generic data analysis tools as well as three tools specifically developed for FLAVIdB: Block entropy analysis, BBscore, and viral species classifier (Table 4).

### Workflows

To accommodate rapid and extensive analysis without the need for local computation or moving data between individual analysis tools or different web servers, FLAVIdB includes pre-defined analysis workflows. A workflow is an automated process that takes a request from the user, performs complex analysis by combining data and tools preselected for common questions, and produces a comprehensive report [13]. These workflows demonstrate both the utility and flexibility of the data mining infrastructure in FLAVIdB. For the current release we have developed and implemented two data mining workflows; a summary workflow and a query analyzer workflow.

### Summary workflow

**Figure 3:**
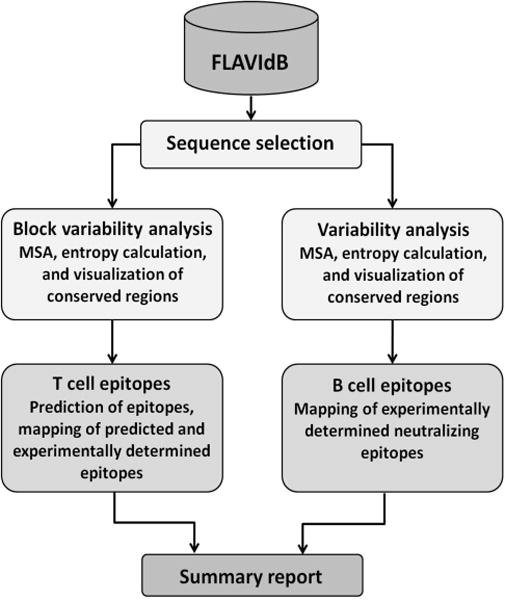
Flowchart of the summary workflow. Initially the user is prompted to select a data set, using a set of filter parameters and/or key word search of the entire database. Then, T-cell epitope prediction, and B-cell epitope characterization are applied.

The summary workflow can be applied to all sequences in the database or to any defined subset thereof. The purpose of this workflow is to summarize potential vaccine targets common to all entries within the FLAVIdB, or a subset of entries, such as summary analysis of one or more species within the FLAVIdB. The results of application of each analysis tool are presented to the user in a single printable output report. The structure and components of the summary workflow are presented in the flowchart in figure 3. The utility of the summary workflow is particularly important at the very beginning of research projects, or as an incremental analysis of existing projects upon the database update.

### Query analyzer workflow

**Figure 4:**
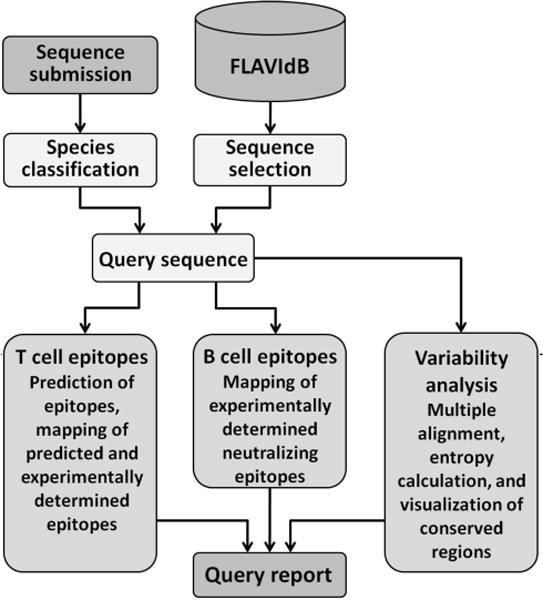
Flowchart of the steps in the query analyzer workflow. The query sequence is selected either from the database or submitted directly through the input window. If manual submission is used, the species is first classified using the species classification tool. T-cell epitopes are predicted and B-cell epitopes are characterized. Finally, a variability analysis is performed for sequences related to the query. The combined results are presented in an output report.

The query analyzer workflow is a useful tool for researchers who need to rapidly analyze newly sequenced strains or previously uncharacterized sequences found in the database. The query analyzer workflow applies the existing data mining modules to the query in a predefined order and the analysis results are presented in a single printable output report. The first step is sequence selection – either directly from the database or the nucleotide sequence followed by species classification. The analysis is followed by parallel application of T-cell epitope, B-cell epitope, and variability analysis, and the final step of report generation. The steps and tools involved in the query analyzer workflow are shown in the flowchart in Figure 4

### Application of summary workflow to DENV

**Figure 5:**
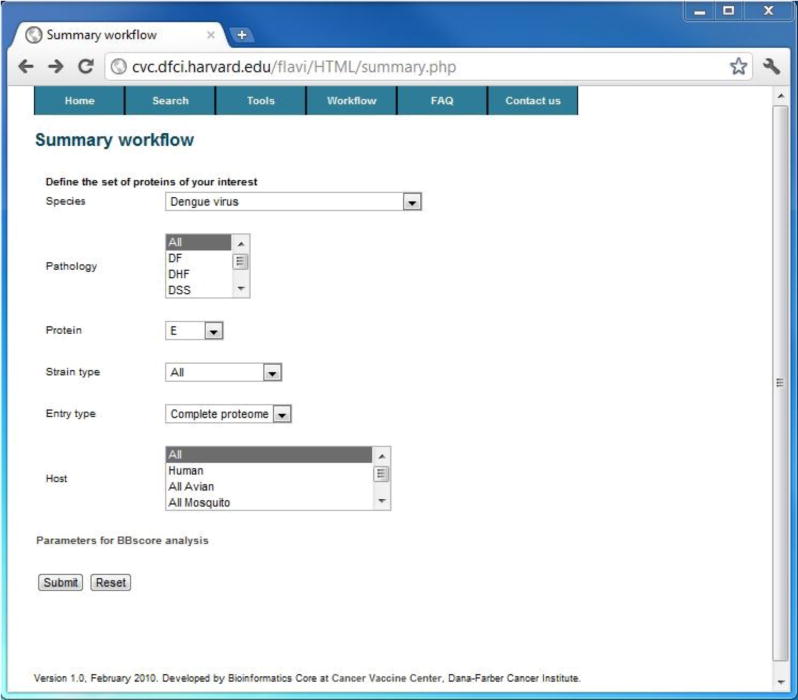
A screenshot of the user input page for the summary workflow.

**Figure 6:**
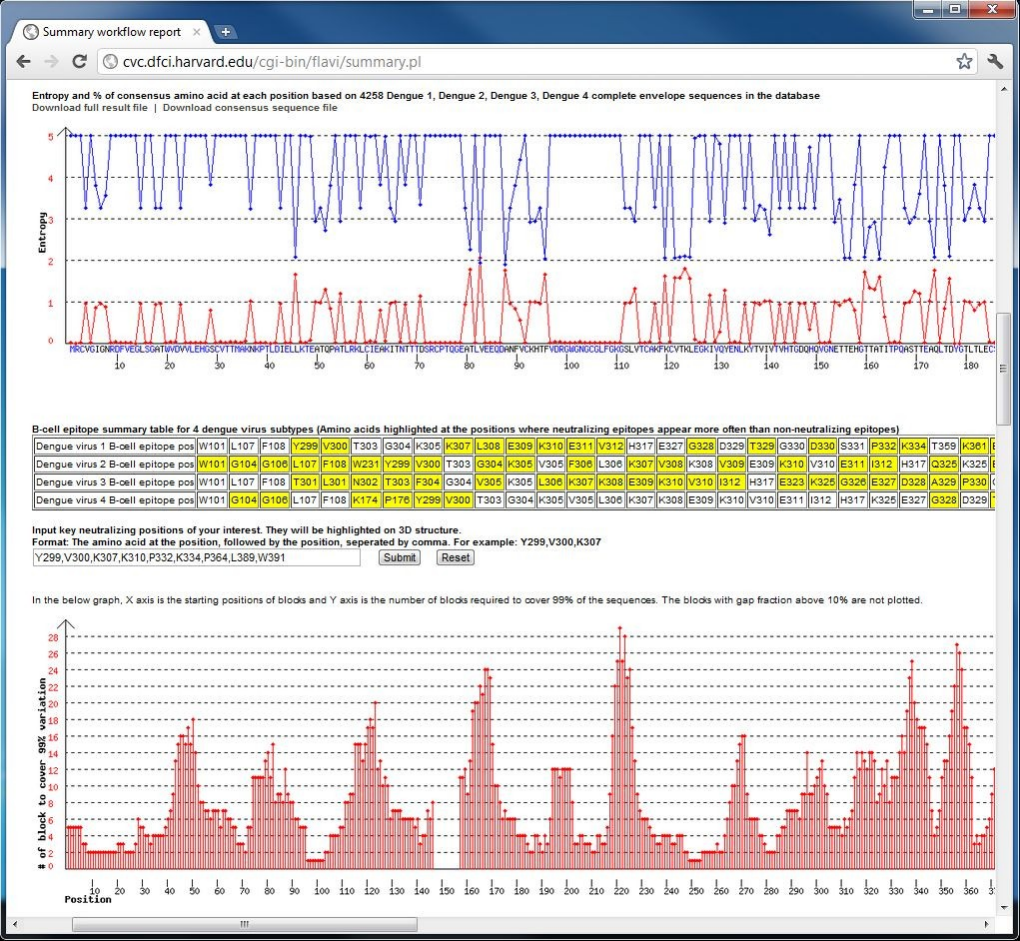
A screenshot of the output report from the summary workflow applied to all four serotypes of DENV. The top graph shows conservation (blue line) and entropy (red line) for the DENV envelope protein, with the consensus amino acids sequence on the x-axis. The following table is a summary of binding assay data and neutralization assay data from IEDB. The bottom graph is the summary of the block entropy analysis. On the x-axis are the starting positions of each peptide block analyzed and on the y-axis is the number of peptides required to achieve an accumulated frequency of 99% within each given block. The lack of data from starting point 148–158 is due to a high fraction of gaps on this position in the multiple sequence alignment.

Application of the summary workflow to all four subtypes of DENV revealed a pool of 333 T-cell epitope candidates, which can potentially be combined in a polyvalent vaccine comprised of five synthetic dengue virus proteins. The analysis of B-cell epitopes revealed five conserved positions in the dengue virus envelope protein that are targeted in antibody-based neutralization. These results were generated by submitting all available DENV sequences to the FLAVIdB summary workflow, thus demonstrating the utility of the work flows in data mining from for comprehensive identification of vaccine targets. Figure 5 shows the input submission screen and Figure 6 shows the conservation and variability of the DENV E protein and the block entropy part of the output report.

## Conclusion

FLAVIdB is a comprehensive database of *Flavivirus* spp. antigens extracted from multiple external sources (GenPept and UniProt, epitope data from IEDB, and structural data from the PDB). The FLAVIdB data has been manually curated and enriched by the extraction of additional annotations available from the corresponding literature, ensuring high data quality and data completeness.

We have integrated the annotated data with the data mining infrastructure consisting of data mining tools for discovery of vaccine targets. This infrastructure provides for an automated data mining platform, enabling extraction of higher level knowledge about T-cell epitopes and neutralizing B-cell epitopes in the flaviviruses. We have also defined and implemented two workflows where data mining tools have been arranged to rapidly and seamlessly extract knowledge from the data stored in the FLAVIdB. The query analyzer workflow enables analysis of single sequences for vaccine targets, whereas the summary workflow summarizes vaccine targets across a larger data set. The modular structure of FLAVIdB can easily be modified for application to other viral pathogens, as well as integration of new analysis modules and workflows.

The main purpose of FLAVIdB is to enable user to perform knowledge discovery from viral antigen data with particular emphasis on applications in immunology and vaccinology. Prediction and characterization of immunogenic epitopes is a critical step in identification and assessment of potential vaccine targets. This process is not identical for different viruses. For example, in DENV it is essential that vaccines are designed to elicit cross-protection to all four serotypes, due to complications in secondary infection by different subtypes. To analyze DENV vaccine targets, is it necessary to identify and compile a very precise and detailed information about antigenic sites which are conserved across all four serotypes, perform a detailed analysis of antigenic diversity, and understand variants representation both in time and geographic spread. In FLAVIdB, the analysis tools are organized in the data mining workflows composed of preselected sets of tools applied to user defined data sets from FLAVIdB. With new knowledge accumulating, we plan to extend the tools and workflows so that precise and detailed analysis can be automated and brought directly to the virologist and vaccinologist workbench.

FLAVIdB differs from existing dedicated flavivirus databases since it offers novel analysis tools (BBscore, block entropy analysis, and flavivirus species classification) specifically developed for the analysis of immunological and vaccinological properties of flaviviruses. These tools, along with generic analysis tools, were implemented in the two workflows providing automated report generation. Furthermore, the sequence data in FLAVIdB is fully annotated with protein cleavage sites, T-cell and B-cell epitopes, which is only partially addressed in some of existing dedicated flavivirus sequence data resources.

FLAVIdB represents a new breed of bioinformatics databases that tightly integrates the content (data), analysis tools, and workflows to enable the automation of complex queries, data mining, and report generation. These “new generation” immunological databases shift focus from retrieval and simple analyses, to complex analyses and extraction of high-level knowledge. We expect this database to serve as a template for the development of advanced integrated bioinformatics infrastructure for vaccinology. The modular structure of FLAVIdB enables easy and straight-forward extension with new modules. The framework can be reused for building vaccineoriented databases of other pathogens such as influenza.

## Figures and Tables

**Figure 2:**
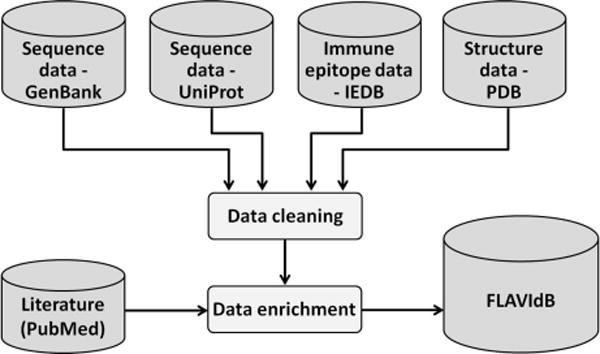
Process of population of the FLAVIdB database with data. The cylinders indicate data repositories and rectangles indicate the manual and semi/automated steps of data cleaning and data enrichment.

**Table 1A:** Most common Flavivirus species in FLAVIdB including their common species abbreviation, number of full proteome sequences, partial proteome sequences, total number of entries, number of T-cell and B-cell epitope entries (note that some epitopes overlap strains and species), and the number of protein structures.

Virus Species	Abbreviation	Full proteome	Partial proteome	Total entries	T-cell epitopes	B-cell epitopes	Structures
Dengue 1	DENV1	1209	1085	2294	112	23	1
Dengue 2	DENV2	835	1781	2616	295	49	1
Dengue 3	DENV3	565	1665	2230	97	40	1
Dengue 4	DENV4	103	606	709	90	24	1
Japanese encephalitis	JEV	67	1238	1305		14	
Kunjin	KUNV	3	103	106			
Kyasanur forest disease	KFDV	2	100	102			
St. Louis encephalitis	SLEV	5	234	239		1	
Tickborne encephalitis	TBE	34	620	654		8	
West Nile	WNV	177	1361	1538	34	38	
Yellow fever	YFV	26	389	415	23	5	

**Table 1B:** Less common *Flavivirus* species in FLAVIdB including their common species abbreviation and total number of entries.

Species	Abbreviation	Entries	Species	Abbreviation	Entries
Murray Valley encephalitis virus	MVEV	70	Cacipacore virus	CPCV	5
Powassan virus	POWV	69	Kumlinge virus	KVE	5
Alkhurma hemorrhagic fever virus	AHFV	36	Tamana bat virus	TAB	4
Omsk hemorrhagic fever virus	OHFV	35	Aroa virus	AROAV	4
Kokobera virus	KOKV	33	Banzi virus	BANV	4
Ilheus virus	ILHV	22	Greek goat encephalitis virus	GGE	4
Louping ill virus	LIV	21	Royal Farm virus	RFV	4
Mosquito flavivirus	MBV	21	Uganda S virus	UGSV	4
Usutu virus	USUV	19	Dakar bat virus	DAKV	4
Aedes flavivirus	MBV	18	Israel turkey meningoencephalomyelitis virus	ITV	4
Tembusu virus	MBV	15	Koutango virus	KOUV	4
Deer Tick virus	DTV	14	Naranjal virus	NJLV	4
Rocio virus	ROC	10	Ntaya virus	NTAV	4
Langat virus	LGTV	9	Sal Vieja virus	SVV	4
Sepik virus	SEPV	9	Bouboui virus	BOUV	3
Edge Hill virus	EHV	9	Potiskum virus	POTV	3
Karshi virus	KSIV	8	Spanish Sheep encephalitis virus	SSE	3
Zika virus	ZIKV	8	Bukalasa bat virus	BBV	3
Entebbe bat virus	ENTV	7	Carey Island virus	CIV	3
Alfuy virus	ALFV	7	Cowbone Ridge virus	CRV	3
Gadgets Gully virus	GGYV	7	Jutiapa virus	JUTP	3
Bagaza virus	BAGV	6	Negishi virus	NIV	3
Wesselsbron virus	WESSV	6	Phnom Penh bat virus	PPBV	3
Modoc virus	MODV	6	Sokoluk virus	SOKV	3
Montana myotis leukoencephalitis virus	MMLV	6	Stratford virus	STRV	3
Bussuquara virus	BSQV	6	Yokose virus	YOKV	3
Saboya virus	SABV	6	Nounane virus	NOUV	2
Kamiti River virus	KRV	5	Chaoyang virus	CYV	2
Kedougou virus	KEDV	5	Turkish Sheep encephalitis virus	TSEV	2
Apoi virus	APOV	5	Batu Cave virus	BCV	2
Rio Bravo virus	RBV	5	Ngoye virus	NGOV	2
Iguape virus	IGUV	5	Yaounde virus	YAOV	2
Jugra virus	JUGV	5	Calbertado virus	CAV	1
Kadam virus	KADV	5	New Mapoon virus	NMV	1
Meaban virus	MEAV	5	San Perlita virus	SPV	1
Saumarez Reef virus	SREV	5	Sitiawan virus	SV	1
Spondweni virus	SPOV	5	T’Ho virus	MBV	1
Tyuleniy virus	TYUV	5	Wang Thong virus	WTV	1

**Table 2:** The data fields in each entry (the values are included if available).

Field name	Field content
**CVC accession**	Accession number unique to FLAVIdB
**GenBank accession**	Accession number unique to GenBank
**Species**	The *Flavivirus* species descriptor
**Type**	Serotype (only applicable to DENV)
**Host**	Host of collection
**Country**	Location of collection
**Year**	Time of collection
**Strain**	Strain name
**Isolate**	Isolate name
**Clone**	Clone name
**Nomenclature**	Short-hand representation of host, country (ISO code), year of collection, serotype (where applicable), strain, isolate, and clone name
**Strain type**	Information on whether strain is wild type, laboratory strain, or vaccine strain
**Pathology**	The morbidity and mortality associated with the virus

**Table 3:** The results of the data enrichment. Direct parsing is performed by considering only information available in the dedicated fields in GenBank entries. GenBank does not have dedicated fields for the information marked with an asterisk (*), so some form of automated text mining was applied to extract this information. The lack of a dedicated field does not necessarily mean that information about strain type, pathology, or sequence annotation is not present in other fields (such as comments/notes) in the entries, but extraction was only possible to automate to a very limited extent without error and artifact propagation.

Type of information	% of entries	
	Direct parsing from GB	Enriched entries in FLAVIdB
Host of collection	49%	72%
Location of collection	83%	96%
Time of collection	53%	84%
Strain type*	6%	72%
Pathology*	0%	3%
Sequence annotation*	30%	100%

**Table 4:** The sources of analysis tools integrated in FLAVIdB and URL for various standalone web servers.

Tool	URL	Reference
BLAST	blast.ncbi.nlm.nih.gov/Blast.cgi	[18]
MAFFT MSA	www.ebi.ac.uk/Tools/msa/mafft/	[17]
NetMHC 3.2	www.cbs.dtu.dk/services/NetMHC/	[23]
NetMHCII 2.2	www.cbs.dtu.dk/services/NetMHCII/	[25]
Species classification	cvc.dfci.harvard.edu/flavi/HTML/species.php	This paper
Block entropy	cvc.dfci.harvard.edu/flavi/HTML/tcell/	This paper
BBscore	cvc.dfci.harvard.edu/flavi/HTML/bcell.php	This paper

## List of abbreviations

ADE: Antibody dependent enhancement
BLAST: Basic local alignment search tool
DENV: Dengue virus
HLA: Human leukocyte antigen
IEDB: Immune epitope database
JEV: Japanese encephalitis virus
MHC: Major histocompatibility complex
MSA: Multiple sequence alignment
PDB: Protein databank
SNP: Single nucleotide polymorphism
TBEV: Tick-borne encephalitis virus
YFV: Yellow fever virus
WNV: West Nile virus
